# Regulation of the translation activity of antigen-specific mRNA is responsible for antigen loss and tumor immune escape in a HER2-expressing tumor model

**DOI:** 10.1038/s41598-019-39557-9

**Published:** 2019-02-27

**Authors:** Baek-Sang Han, Sunhee Ji, Sungwon Woo, Ji Heui Lee, Jeong-Im Sin

**Affiliations:** 1 BIT Medical Convergence Graduate Program and Department of Microbiology, School of Medicine, Kangwon National University, Chuncheon, Gangwon-do 24341 Korea; 2 Department of Anesthesia and Pain Medicine, Korea Cancer Center Hospital, 75 Nowon-ro, Nowon-gu, Seoul, 01812 Korea; 3 Present Address: Gyeonggi-do Institute of Health and Environment, Suwon, Gyeonggi-do 16205 Korea

## Abstract

Tumor cells tend to behave differently in response to immune selective conditions. Contrary to those in therapeutic antitumor conditions, tumor cells in prophylactic antitumor conditions lose antigen expression for antitumor immune escape. Here, using a CT26/HER2 tumor model, we investigate the underlying mechanism(s). We selected tumor cell variants (CT26/HER2-A1 and -A2) displaying resistance to antitumor protective immunity and loss of HER2 antigen expression. These immune-resistant cells failed to induce Ag-specific IgG and IFN-γ responses while forming tumors at the same rate as CT26/HER2 cells. RT-PCR, qRT-PCR, PCR, Western blot and DNA sequencing analyses demonstrated that HER2 expression was inhibited at the post-transcriptional level in these immune-resistant cells, suggesting that tumor cells may escape antitumor immunity through the post-transcriptional regulation of antigen gene expression. The proteasome and lysosomal protein degradation pathways were not responsible for antigen loss, as determined by an inhibitor assay. Finally, HER2 mRNA was found to be not present in the monosomes and polysomes of CT26/HER2-A2 cells, as opposed to CT26/HER2 cells, suggesting that the translation activity of HER2 mRNAs may be suppressed in these immune-resistant cells. Taken together, our results report a new mechanism by which tumor cells respond to antitumor protective immunity for antitumor immune evasion.

## Introduction

Human epidermal growth factor receptor 2 [HER2] (also known as Her-2/neu and erbB-2), as an oncogenic protein, has an important function in the development of breast cancer^1,2^. Besides breast cancer cells, ovarian and colorectal cancer cells also express high levels of HER2^3,4^. HER2-positive breast cancers tend to be more aggressive and to spread more quickly than HER2-negative breast cancers^3^. For instance, 5 year survival rates and recurrence rates of patients with HER2-positive breast cancer are far higher than those of patients with HER2-negative breast cancer. This makes the HER2 levels useful for predicting therapeutic outcomes in breast cancer patients. In HER2-positive cancer patients, antibodies and T cells specific for HER2 are detectable^5,6^. In this context, HER2 proteins have been used as therapy target for patients with HER2-positive cancers.

Tumor-specific CTLs have been known to play a critical role in tumor cell lysis in antitumor immunotherapy. In a recent report, HER2_63-71_-specific CD8+ CTLs are responsible for tumor regression in the 4T1.2/HER2 and CT26/HER2 models^7^ and in a mouse mammary tumor (D2F2/E2 expressing HER2) model^8^. HER2 DNA vaccines elicited Ag-specific CTL responses, leading to tumor protection^9^. A major role of CTLs in tumor eradication has also been reported in other tumor models, such as TC-1, B16 and MC32^10–12^. Despite this, numerous evidence has shown that tumor cells counter antitumor CTL immunity by losing their antigen or MHC class I molecules^13,14^. Similar to this, we also observed that tumor cells acquired Ag-specific CTL resistance through the loss of tumor antigen in the MC32 tumor prophylactic model^15^. In the MC32 tumor therapeutic model, on the other hand, tumor cells acquired CTL resistance through losing antigen presentation in conjunction with MHC class I molecules^12^. It is likely that the tumor cells of the prophylactic tumor model escape Ag-specific CTL-mediated surveillance somewhat differently from those of the therapeutic tumor model. Tumor cells are also known to produce immune inhibitory molecules (such as galectin-9, transforming growth factor-β, indoleamine 2,3-dioxygenase, serine protease inhibitor, etc.) for the inhibition of Ag-specific CTLs^16–19^. It has also been reported that immune selection pressures allow tumor cells to develop stem-like phenotypes with CTL resistance in the TC-1 model^20^. In this context, it is likely that antitumor immunity may serve as a biological selective pressure that promotes the emergence of immune escape tumor cell variants, as suggested by Schreiber’s group^21^. Moreover, clarification of altered biological functions of tumor cells for antitumor CTL escape is likely important for understanding tumor cell’s behavior under various immune selective conditions.

In this study, we observed in a prophylactic CT26/HER2 tumor model that despite their CTL induction status, a few mice formed tumors when they were challenged with a high number of tumor cells. To clarify how these tumor cells acquired immune escape functions, we obtained tumor cells from tumor-formed immune mice, and designated them as CT26/HER2-A1 and -A2 cells. CT26/HER2-A1 and -A2 tumor cells failed to express HER2, lost the capacity to stimulate Ag-specific immune cells and remained insensitive to antitumor immunity by forming tumors in HER2-immune mice. These tumor cells lost antigen expression at the post-transcriptional level, leading to antitumor immune evasion. Moreover, the loss of tumor antigen was found to be mediated by inhibiting the translational activity of its mRNAs, but not through the modification of protein degradation pathways. This is a new finding that immune selection pressure may allow tumor cells to inhibit the translation activity of their antigen mRNAs at the post-transcriptional level and that the loss of tumor antigen is responsible for tumor immune escape.

## Results

### Immune-stimulating activity and antitumor immune sensitivity remained absent in tumor cells from tumor-bearing immune mice in the prophylactic tumor model

**Figure 1:**
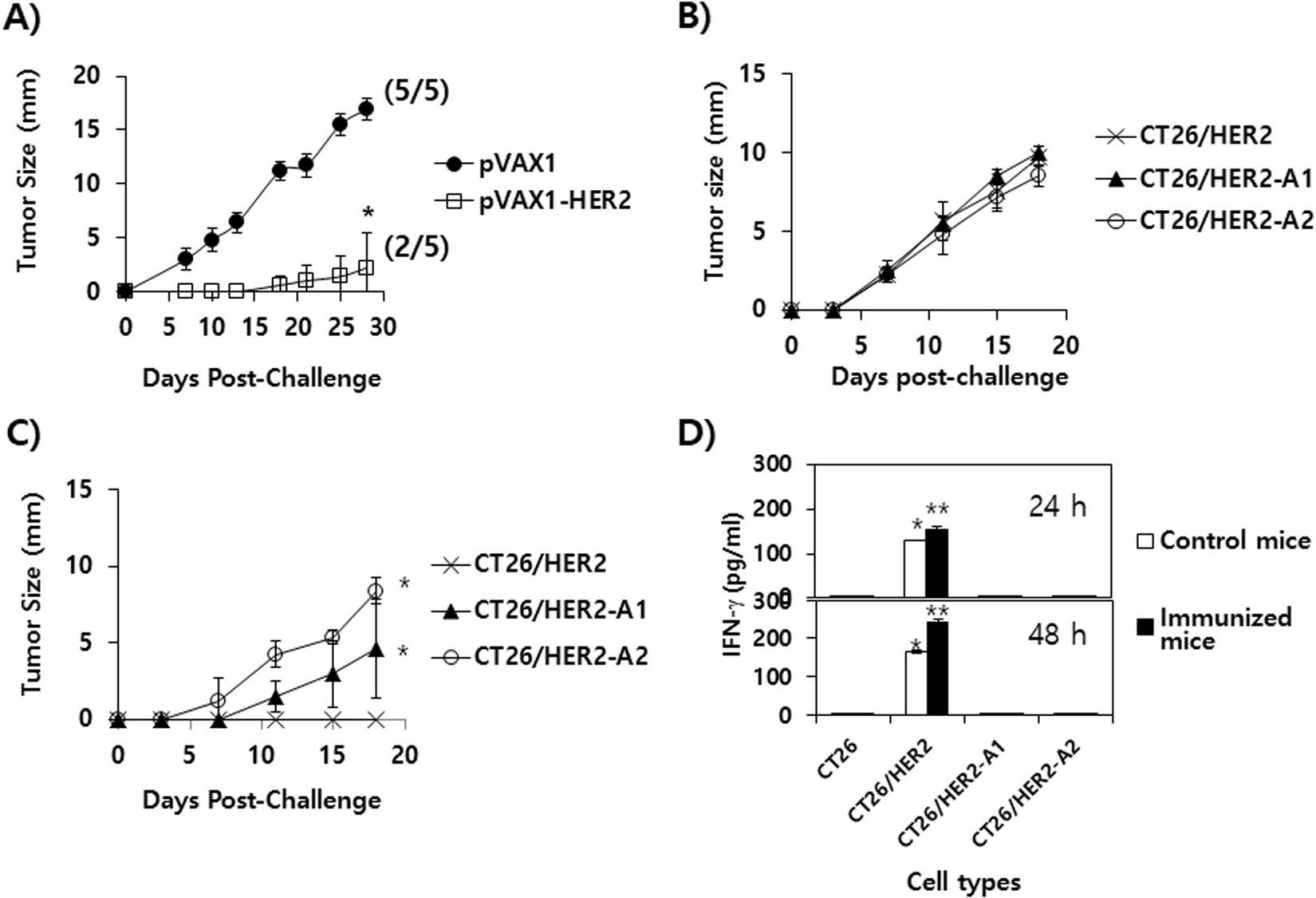
Formation of tumors by a challenge with CT26/HER2 tumor cells in HER2 DNA vaccine-immunized mice (**A**), and the tumor-forming (**B**,**C**) and immune cell stimulating (**D**) activity of CT26/HER2, CT26/HER2-A1 and CT26/HER2-A2 cells. (**A**) Mice (n = 5/group) were immunized at 0 and 1 weeks by IM-EP with 50 μg of HER2 DNA vaccines. At 2 weeks, the mice were challenged with 1 × 10^6^ CT26/HER2 cells per mouse. The tumor sizes were measured over time. The values and bars indicate mean tumor sizes and the SD, respectively. The values in (/) represent the number of mice displaying tumors at 28 days post-challenge/the number of mice tested. *p < 0.05 compared to pVAX1. (**B**,**C**) Each of the naïve (**B**, n = 4/group) and HER2 DNA vaccine-immunized mice (**C**, n = 4/group) was challenged s.c. with 3 × 10^5^ CT26/HER2 (upper left flank), CT26/HER2-A1 (lower left flank) and CT26/HER2-A2 (upper right flank) cells per mouse. HER2 DNA vaccine-immunized mice indicate the mice that were injected at 0 and 1 weeks by IM-EP with 50 μg of HER2 DNA vaccines and then re-immunized by IM-EP with HER2 DNA vaccines one week prior to a challenge with 3 different tumor cell types. The tumor sizes were measured over time. The values and bars indicate mean tumor sizes and the SD, respectively. *p < 0.05 compared to CT26/HER2. (**D**) Each of UV-exposed tumor cells (5 × 10^5^), CT26/HER2, CT26/HER2-A1 and CT26/HER2-A2 was incubated for 1 and 2 days with 6 × 10^6^ immune cells from naïve and HER2 DNA vaccine-immunized mice. HER2 DNA vaccine-immunized mice were obtained by injecting the mice with 50 μg of HER2 DNA vaccines by IM-EP, followed by a booster injection at 1 week following the first injection. One week after the final immunization, the mice were sacrificed to obtain immune cells. The cell supernatants were collected after 1 and 2 days of cell culture for an IFN-γ assay. The values and bars indicate mean IFN-γ amounts and the SD, respectively. *p < 0.05 compared to CT26. **p < 0.05 compared to control mice.

We previously reported that HER2 DNA vaccines could induce HER2_63-71_-specific CTL responses and complete antitumor protection from a challenge with 5 × 10^5^ cells per mouse^9^. In this study, we challenged immunized mice with a higher number of tumor cells (1 × 10^6^ per mouse). This experiment was performed to determine how the Ag-specific CTLs might respond to a higher number of tumor cells upon challenge. The data from Fig. 1A showed that 2 of the 5 HER2 DNA vaccine-immunized mice formed tumors, which grew thereafter. This result indicates that, when challenged with a higher number of CT26/HER2 tumor cells, tumor cells tend to acquire the ability to resist antitumor immunity induced by HER2 DNA vaccination, leading to tumor formation. To test this possibility, we surgically removed tumor tissues from the 2 tumor-bearing mice at 28 days post-challenge (from Fig. 1A) in a prophylactic setting. After more than 5 rounds of tumor cell culture *in vitro*, we designated the cells as CT26/HER2-A1 and CT26/HER2-A2 cells. To test whether these tumor cells might form tumors in mice with antitumor immunity, each of the immune mice was challenged with wild type CT26/HER2 cells on the upper left flank, CT26/HER2-A1 cells on the upper right flank and CT26/HER2-A2 cells on the lower right flank. All 3 types of tumor cells were able to form tumors in naïve mice (Fig. 1B). Moreover, CT26/HER2-A1 and CT26/HER2-A2 cells formed tumors that continued to grow in all of the tested immune mice (Fig. 1C). In contrast, wild type CT26/HER2 cells failed to form tumors in all of the tested immune mice. These data indicate that CT26/HER2-A1 and -A2 cells are resistant to HER2-specific CTL-mediated tumor cell killing. We next tested whether these tumor cells might possess the capacity to stimulate immune cells isolated from naïve and HER2 vaccine-immunized mice *in vitro*. When UV-exposed wild type CT26/HER2 cells were used as stimulating agents for immune cells from naïve and HER2 vaccine-immunized mice, they induced IFN-γ production from both groups of immune cells (Fig. 1D). In contrast, CT26 cells without HER2 expression failed to induce IFN-γ production from both types of immune cells. Immune cells from naïve mice also induced IFN-γ production when they were stimulated *in vitro* with tumor cells expressing human HER2 antigens, suggesting that this IFN-γ production might result from a xenogeneic reaction. Furthermore, immune cells isolated from HER2 vaccine-immunized mice produced significantly more IFN-γ than those from naïve mice, indicating that this increased amount of IFN-γ might be produced from Ag-specific T cells that were induced by HER2 DNA vaccines. When UV-exposed CT26/HER2-A1 and CT26/HER2-A2 cells were used as stimulating agents for immune cells from naïve and HER2 vaccine-immunized mice, they were unable to produce IFN-γ from both types of immune cells. These results imply that HER2 antigens alone and in conjunction with MHC class I molecules may be lacking on the surface of CT26/HER2-A1 and CT26/HER2-A2 cells, which might be responsible for the lack of IFN-γ induction from immune cells as well as the lack of tumor control in HER2 vaccine-immunized mice.

### Expression status of MHC class I, HER2 antigens and immune inhibitory ligands, as well as CD80, on the cell surface of CT26/HER2, CT26/HER2-A1 and CT26/HER2-A2 cells

**Figure 2:**
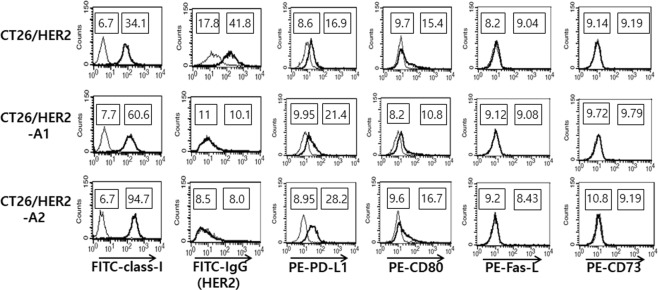
Expression levels of MHC class I (H-2K^d^), HER2, PD-L1, CD80, Fas-L, and CD73 molecules on the surface of CT26/HER2, CT26/HER2-A1 and CT26/HER2-A2 cells. The tumor cells (1 × 10^6^) were incubated with FITC/PE-conjugated Abs specific for MHC class I, PD-L1, CD80, Fas-L and CD73. Thin line, FITC- or PE-conjugated control Abs; thick line, FITC- or PE-conjugated Abs specific for class I, PD-L1, CD80, Fas-L and CD73. For staining HER2 antigens, anti-HER2 sera obtained from HER2 DNA vaccine-immunized mice and naïve sera as a control were used as primary Abs, followed by reaction with FITC-conjugated anti-mouse IgG Abs as the secondary Abs. Thin line, naïve sera; thick line, anti-HER2 sera. The numbers in the left square indicate the mean fluorescence intensity (MFI) values of control Abs or naïve sera (thin line) while those in the right square indicate the MFI values of experimental Abs or anti-HER2 sera (thick line). This was repeated with similar results.

It is known that immune inhibitory molecules (PD-L1, Fas-L and CD73) are associated with inhibition of tumor-specific T cell activity^22–24^. In addition, the expression status of antigen, MHC class I and CD80 molecules is associated with the regulation of Ag-specific T cell responses. In this context, we evaluated the expression levels of MHC class I, HER2, PD-L1, CD80, Fas-L and CD73 molecules on the surface of CT26/HER2, CT26/HER2-A1 and CT26/HER2-A2 cells. As shown in Fig. 2, all of the tested tumor cells expressed MHC class I molecules. However, wild type CT26/HER2 cells expressed HER2 antigens, while CT26/HER2-A1 and CT26/HER2-A2 cells did not. Thus, it is highly likely that the lack of HER2 antigens in CT26/HER2-A1 and CT26/HER2-A2 cells might be responsible for their resistance to antitumor immunity driven by HER2 DNA vaccination. When tumor cells were tested for the expression levels of the immune inhibitory ligands, there was no significant difference in the expression levels of Fas-L and CD73 among these tumor cells, suggesting that these inhibitory molecules are not associated with resistance to antitumor immunity in this setting. In particular, CT26/HER2-A1 and CT26/HER2-A2 cells displayed an increased level of MHC class I and PD-L1 expression on the cell surface, as compared to CT26/HER2 cells. Taken together, these results show that CT26/HER2 cells can acquire the ability to form tumors in immune mice by losing antigen expression in the prophylactic setting, which contributes them to grow even in the presence of Ag-specific CTL lytic activity.

### Evaluation of the abilities of CT26/HER2 cells vs. CT26/HER2-A1 and -A2 cells to grow and induce Ag-specific antibody responses *in vivo*

**Figure 3:**
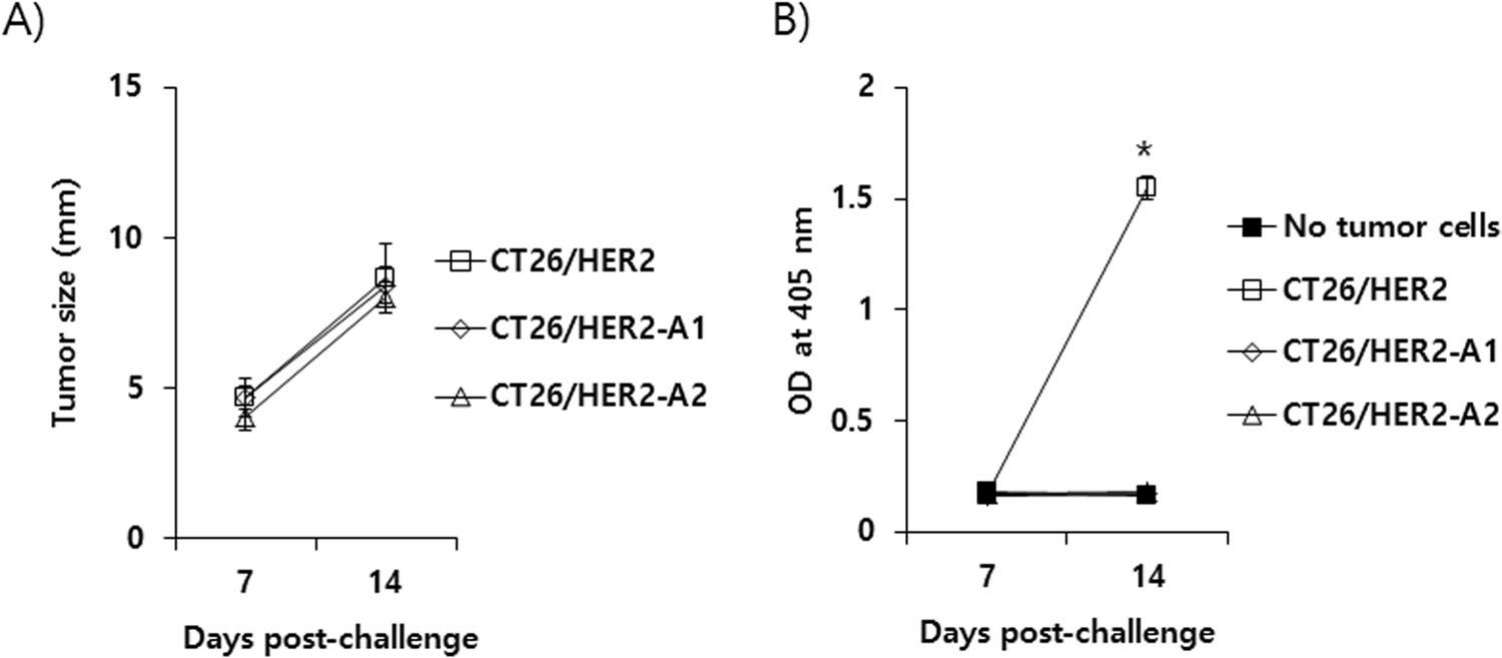
Evaluation of tumor sizes (**A**) and HER2-specific antibody levels (**B**) in mice challenged with CT26/HER2, CT26/HER2-A1 and CT26/HER2-A2 cells. (**A**,**B**) Each group of mice (n = 5/group) was challenged s.c. with 5 × 10^5^ CT26/HER2 cells, CT26/HER2-A1 cells and CT26/HER2-A2 cells per mouse. Tumor sizes were measured at 7 and 14 days post-tumor cell challenge (**A**). At the same time, the mice were bled and sera were equally pooled. The sera were diluted and used to measure HER2-specific antibody levels by ELISA (**B**). *p < 0.05 compared to no tumor cells (naïve sera).

We evaluated the abilities of CT26/HER2 cells vs. CT26/HER2-A1 and -A2 cells to grow and induce Ag-specific antibody responses in naive mice. As shown in Fig. 3A, CT26/HER2, CT26/HER2-A1, and CT26/HER2-A2 cells grew at the same rate when their tumor sizes were measured at 7 and 14 days following a tumor cell challenge. When Ag-specific antibody levels were tested, CT26/HER2 cells induced Ag-specific antibody responses, whereas CT26/HER2-A1 and -A2 cells failed to induce Ag-specific antibody responses (Fig. 3B). Taken together, these data show that CT26/HER2-A1 and -A2 cells grow at the same rate as CT26/HER2 cells but are unable to induce HER2 (a xenogeneic antigen)-specific antibody responses, possibly resulting from a lack of HER2 proteins on the surface of these cells. This result supports the notion that CT26/HER2-A1 and -A2 cells do not express HER2 proteins on the cell surface.

### HER2 DNA, RNA and proteins and their regulation in CT26/HER2, CT26/HER2-A1, and CT26/HER2-A2 cells

**Figure 4:**
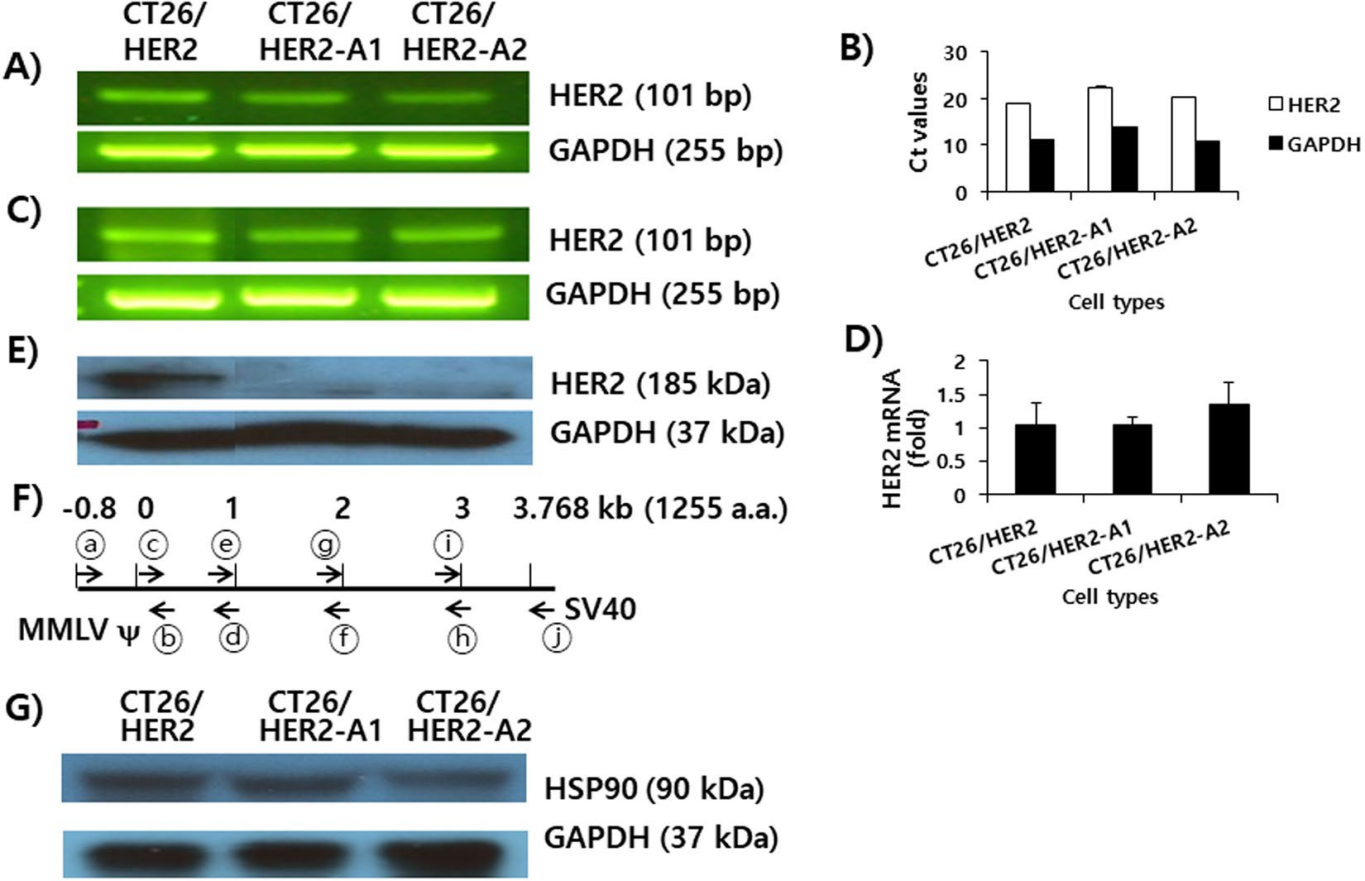
The levels of HER2 DNA (**A**,**B**), mRNA expression (**C**,**D**), and protein expression (**E**) in CT26/HER2, CT26/HER2-A1 and CT26/HER2-A2 cells, along with DNA sequencing schemes (**F**) and the expression status of HSP90 (**G**). (**A**) The tumor cells were lysed for genomic DNA purification. Four hundred nanograms of genomic DNA were tested in a PCR assay for HER2, as described in the Materials and Methods. (**B**) Four hundred nanograms of genomic DNA were also tested using qRT-PCR assay. Ct values are defined as the number of cycles required for the fluorescent signal to cross the threshold. (**C**) The tumor cells were treated with TRIzol and then the RNA was isolated, followed by RT-PCR assay. (**D**) cDNA (generated by RT) was also tested using qRT-PCR assay. The data were used to calculate the levels of HER2 mRNA expression, as described in the Materials and Methods. (**E**) The tumor cells were lysed in RIPA buffer, and 30 μg samples of the cell lysates were separated by SDS-PAGE and analyzed by Western blot assay against HER2 proteins, as described in the Materials and Methods. (**F**) Shows the whole HER2 gene regions of genomic DNAs and their upstream and downstream regions from CT26/HER2-A1 and -A2 cells that were amplified by PCR using forward and reverse primers (arrows). The PCR products were purified and sequenced. (**G**) Similar experiments to those shown in (**E**) except using anti-HSP90 antibodies for Western blot assay.

As CT26/HER2-A1 and -A2 cells failed to express HER2 antigens, we tested whether HER2 genes might be lost from the genome of these cells. The polymerase chain reaction (PCR) data in Fig. 4A demonstrated that HER2 genes were present in the genomic DNA of CT26/HER2-A1 and -A2 cells. Based upon the observed Ct values (<29) in quantitative real-time (qRT)-PCR analysis (Fig. 4B), HER2 gene targets were also abundant in these tumor cells. Also, HER2 mRNAs were present in CT26/HER2-A1 and -A2 cells at quantities similar to those in CT26/HER2 cells, as determined by reverse transcription (RT)-PCR assays (Fig. 4C). This result is supported by the data of qRT-PCR assay displaying similar levels of HER2 mRNA expression in these tumor cells (Fig. 4D). In this study, we also tested whether the HER2 mRNA detection might be due to any contaminated genomic DNAs in the tested RNA samples. In our PCR assay, however, we detected no HER2 bands from the RNA samples tested prior to RT reaction (data not shown), confirming that the HER2 bands are indeed from HER2-specific mRNAs. In Western blot assay, however, CT26/HER2-A1 and -A2 cells did not express a 185 kDa of HER2 proteins (Fig. 4E). In contrast, CT26/HER2 cells expressed HER2 proteins. These data suggest that CT26/HER2-A1 and -A2 cells may resist antitumor immunity through the loss of antigen expression. As both DNA and mRNA specific for HER2 proteins, but not HER2 proteins, were detectable in CT26/HER2-A1 and -A2 cells, we reasoned that HER2 genes might have obtained some genetic mutation, thereby leading to the translational truncation of HER2 proteins. To test this possibility, we sequenced whole HER2 DNA sequences in CT26/HER2 cells, as well as CT26/HER2-A1 and -A2 cells. For DNA sequencing analysis (Fig. 4F), we utilized the primers (forward primers ⓐ; 5′-TTTTTGTGGCCCGACCTGAG-3′ spanning Molony murine leukemia virus [MMLV] ψ regions, reverse primers ⓑ; 5′-CAAGAGGGCGAGGAGGAG-3′ spanning HER2 DNA sequences 33-50) for sequencing the early HER2 gene sequences and their upstream sequences, and the primers [first HER2 fragment (forward primers ⓒ; 5′-ATGGAGCTGGCGGCCTTGTG-3′ spanning HER2 gene sequences 1–20, reverse primer ⓓ: 5′-CTTCTCACACCGCTGTGTTCC-3′ spanning HER2 gene sequences 980–999), second HER2 fragment (forward primers ⓔ; 5′-TGCACAACCAAGAGGTGACA-3′ spanning HER2 gene sequences 950–970, reverse primer ⓕ: 5′-CGCTTGATGAGGATCCCAAAG-3′ spanning HER2 gene sequences 2010–2030), third HER2 fragment (forward primers ⓖ; 5′-GTGGTTGGCATTCTGCTGGT-3′ spanning HER2 gene sequences 1972–1991, reverse primer ⓗ: 5′-AGTCCTCATTCTGGATGACCA-3′ spanning HER2 gene sequences 2960–2980), and forth HER2 fragment (forward primers ⓘ; 5′-GGAGTTGGTGTCTGAATTCTC-3′ spanning HER2 gene sequences 2910–2930, reverse primer ⓙ: 5′-ACCCTAACTGACACACATTCC-3′ spanning the SV40 promoter region)] for sequencing whole HER2 genes and their downstream sequences. The nucleotide and amino acid sequences of the whole HER2 DNA had no genetic mutations (data not shown). These data suggest that HER2 cDNA is intact in CT26/HER2-A1 and -A2 cells. Furthermore, CT26/HER2-A1 and -A2 cells failed to induce IFN-γ when they were used as stimulating agents for immune cells from HER2 vaccine-immunized mice (Fig. 1D). These data indicate that CT26/HER2-A1 and -A2 cells post-transcriptionally inhibit antigen expression. Taken together, these results suggest that the immune selective pressure imposed by HER2 DNA vaccination might allow tumor cells to post-transcriptionally inhibit antigen expression, resulting in tumor cell immune escape.

### The loss of HER2 protein expression was not mediated by heat shock protein (HSP)90 and the protein degradation pathways

**Figure 5:**
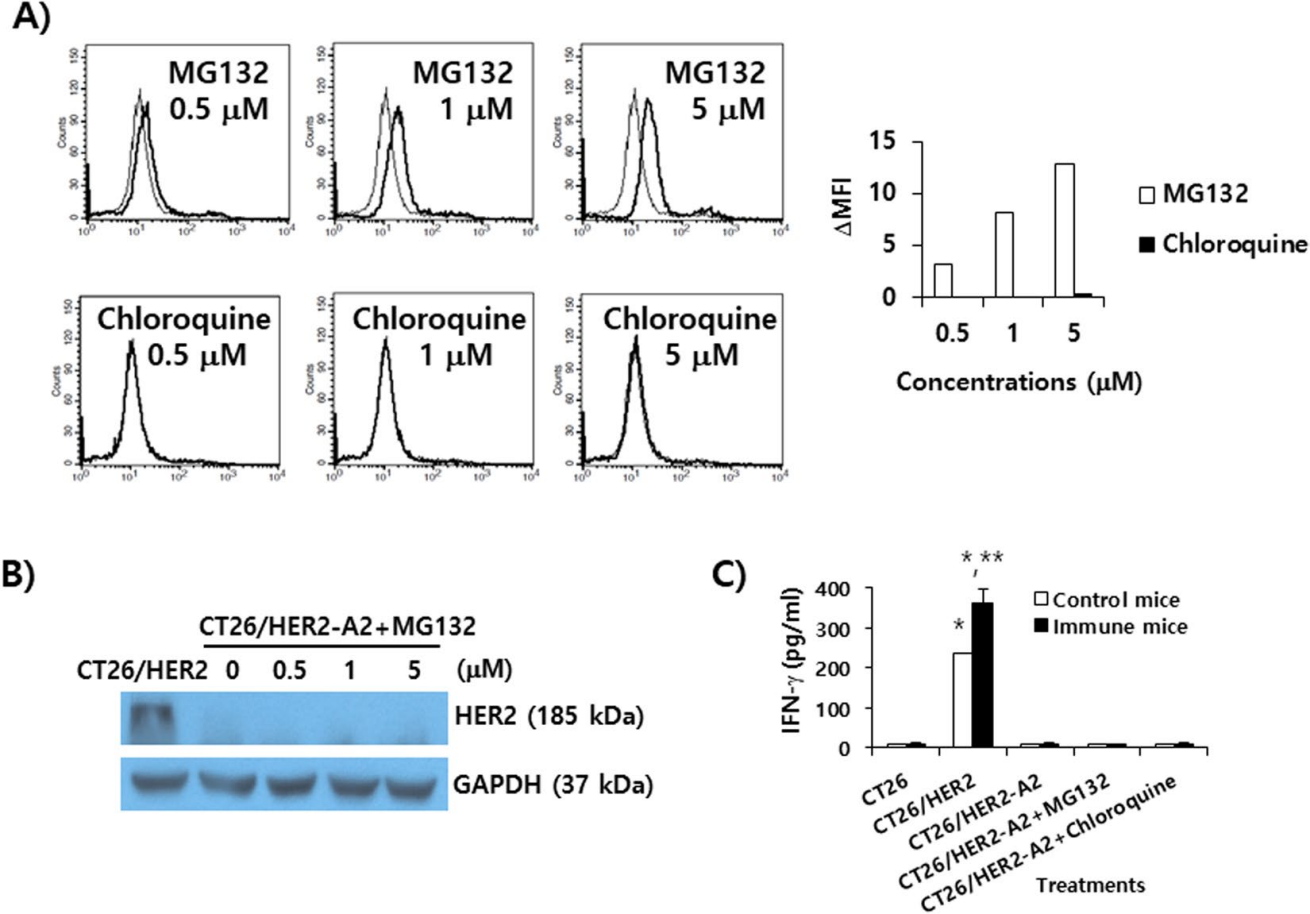
Evaluation of HER2 expression and of Ag-specific immune stimulatory activity in CT26/HER2-A2 cells by treatment with MG132 or chloroquine. (**A**) CT26/HER2-A2 cells were incubated for 1 day with an increasing concentration of MG132 and chloroquine. The cells were reacted with 2 µl of anti-HER2 sera and then stained with FITC-conjugated anti-mouse IgG for flow cytometry. Thin line; non-treatment, thick line; drug treatment. Anti-HER2 sera were obtained from mice immunized twice with HER2 DNA vaccines. ΔMFI was calculated as [the MFI values of drug treatment - the MFI values of non-treatment]. (**B**) CT26/HER2-A2 cells were incubated for 1 day with an increasing concentration of MG132. The tumor cells were lysed in RIPA buffer, and 30 μg samples of the cell lysates were separated by SDS-PAGE and analyzed by Western blot assay. (**C**) Mice were immunized by IM-EP with HER2 DNA vaccines at 0 and 1 weeks. At 2 weeks, the mice were sacrificed to obtain splenocytes. The splenocytes were incubated for 2 days with CT26/HER2-A2 cells that had been treated for 1 day with 5 µM MG-132 and chloroquine and then exposed to UV light for 3 h prior to immune cell treatment. The cell supernatants were collected for IFN-γ assay. *p < 0.05 compared to CT26. **p < 0.05 compared to control mice.

HSP90 (a chaperone protein) has been known to regulate the stability and functions of numerous oncogenic proteins^25^. We tested whether CT26/HER2-A1 and -A2 cells might have a defect in HSP90 expression. As seen in Fig. 4G, there was no significant difference in the expression levels of HSP90 between CT26/HER2 cells and CT26/HER2-A1 and -A2 cells. This result suggests that HSP90 may not be associated with the loss of HER2 expression in these immune-resistant cells. Next, we speculated that the loss of HER2 expression might be mediated by protein degradation pathways in CT26/HER2-A1 and -A2 cells. To test this possibility, we chose CT26/HER2-A2 cells, and treated them with either MG132 (as inhibitors of the proteasome degradation pathway) or chloroquine (as inhibitors of the lysosomal degradation pathway). As shown in Fig. 5A, MG132 treatment increased HER2 expression levels in a drug dose-dependent manner, as measured by flow cytometry. In the case of chloroquine treatment, there was no increase in HER2 expression levels on the cell surface. To confirm these data, we performed Western blot assay. As shown in Fig. 5B, CT26/HER2-A2 cells had no HER2 protein expression by treatment with MG132. However, wild type CT26/HER2 cells expressed HER2 proteins. In this case, the difference in HER2 expression status between flow cytometry and Western blot assay might be due to the artificial effects of MG132-treated cells in flow cytometry. This is supported by our subsequent finding that fluorescence-positive cells were still detectable when the cells were tested using flow cytometry even in the absence of any reactions with HER2-specific primary Abs and fluorochrome-conjugated secondary Abs (data not included), suggesting that DG132 treatment may cause cells to generate auto-fluorescence. In parallel with this, CT26/HER2-A2 cells were unable to restore the capacity to stimulate immune cells by treatment with MG132 or chloroquine (Fig. 5C). In contrast, CT26/HER2 cells induced both xenogeneic and HER2 vaccine-induced T cell-specific IFN-γ production from immune cells. Thus, these results show that HSP90 and protein degradation pathways are not responsible for the loss of HER2 protein expression in these tumor cells.

### The effects of various cytotoxic drugs on the HER2 expression and immune stimulatory activity of CT26/HER2-A2 cells

**Figure 6:**
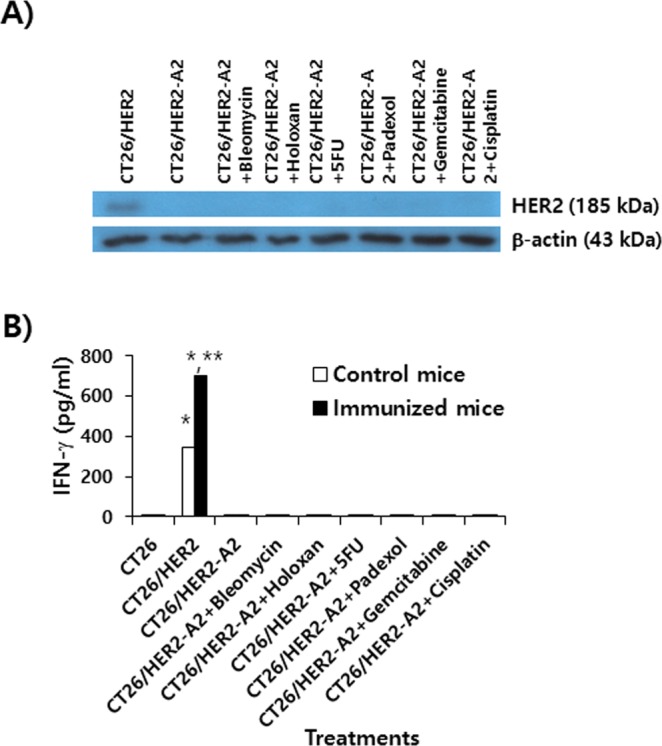
Evaluation of HER2 expression and of Ag-specific immune stimulatory activity in CT26/HER2-A2 cells by treatment with cytotoxic drugs. (**A**) CT26/HER2-A2 cells were incubated for 1 day with 1 μg/ml of drugs (5-FU, gemcitabine, cisplatin, holoxan, bleomycin, padexol). The tumor cells were lysed in RIPA buffer, and 30 μg samples of the cell lysates were separated by SDS-PAGE and analyzed by Western blot assay. (**B**) Mice were immunized by IM-EP with HER2 DNA vaccines at 0 and 1 weeks. At 2 weeks, the mice were sacrificed to obtain splenocytes. The splenocytes were incubated for 2 days with CT26/HER2-A2 cells that had been treated for 1 day with 1 µg of each drug per ml and then exposed to UV light for 3 h prior to immune cell treatment. The cell supernatants were collected for IFN-γ assay. *p < 0.05 compared to CT26. **p < 0.05 compared to control mice.

It has been known that acquisition of antitumor drug resistance is tightly regulated by RNA-binding proteins (RBPs) and miRNAs in tumor cells (reviewed in^26^). In this regard, we speculated that treatment of CT26/HER2-A2 cells with various chemotherapeutic drugs might alter the fates of RBPs and miRNAs, possibly leading to HER2 recovery. To test this possibility, we examined whether CT26/HER2-A2 cells following treatment with bleomycin, holoxan, 5-FU, padexol, gemcitabine, and cisplatin might have acquired both HER2 proteins and their immune stimulatory activity. We first tested this hypothesis using Western blot assay. As shown in Fig. 6A, CT26/HER2-A2 cells treated with bleomycin, holoxan, 5-FU, padexol, gemcitabine, and cisplatin did not express HER2 proteins (185 kDa). In contrast, wild type CT26/HER2 cells expressed HER2 protein (185 kDa). In parallel with this, CT26/HER2-A2 cells treated with bleomycin, holoxan, 5-FU, padexol, gemcitabine, and cisplatin were unable to restore the capacity to stimulate both naïve and HER2-specific immune cells (Fig. 6B). In contrast, wild type CT26/HER2 cells induced both xenogeneic and HER2 vaccine-induced T cell-specific IFN-γ production from immune cells. This result suggests that the tested chemotherapeutic drugs may not have any effects on the restoration of HER2 antigen expression in these immune evasive cells.

### HER2 mRNAs were not present in the monosomes and polysomes of CT26/HER2-A2 cells, as opposed to CT26/HER2 cells

**Figure 7:**
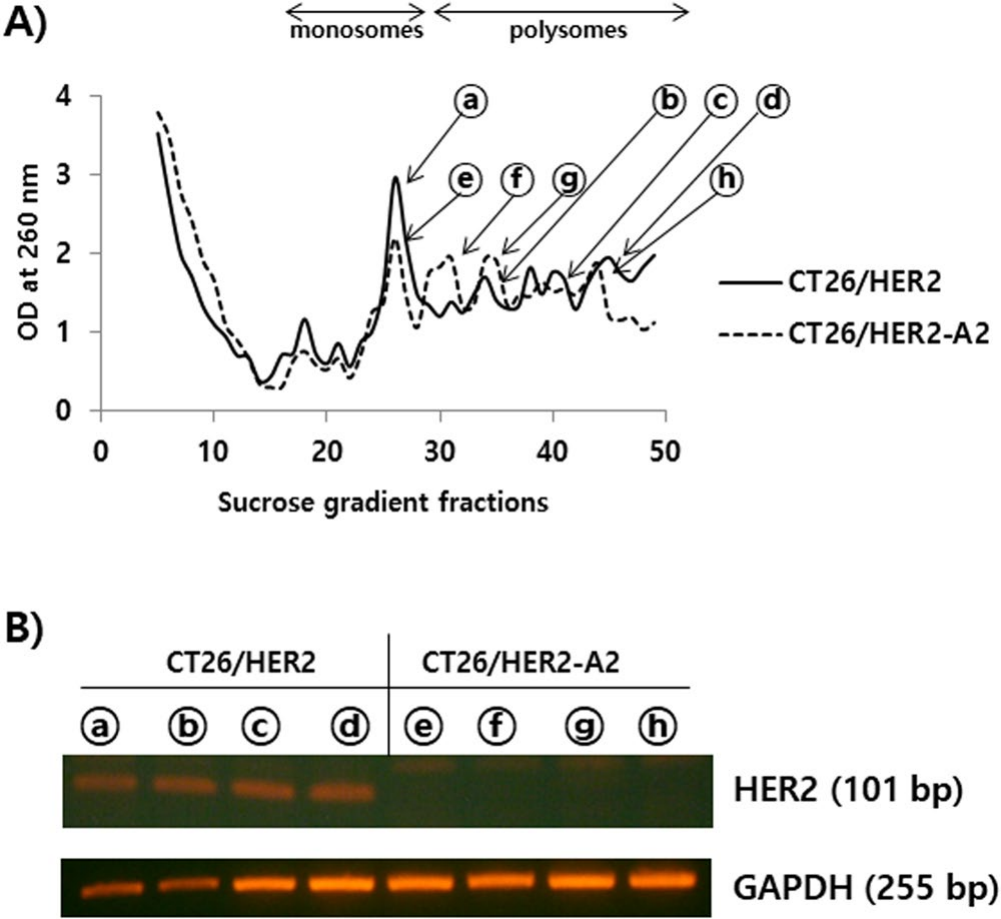
Evaluation of HER2 mRNA levels in the monosomes and polysomes of CT26/HER2 cells vs. CT26/HER2-A2 cells. (**A**) CT26/HER2 and CT26/HER2-A2 cells were grown to 80–90% confluency. The cells were harvested to obtain cell lysates, as described in the Materials and Methods. The cells lysates were loaded to sucrose gradients and centrifuged. The OD values of each collected fraction were graphed. The selected fractions containing the monosomes and polysomes of CT26/HER2 and CT26/HER2-A2 cells are indicated by arrows. (**B**) The selected fractions were treated with TRIzol and RNAs were isolated, followed by RT-PCR, as described in the Materials and Methods.

Next, we speculated that CT26/HER2-A2 cells might have obtained a defect in HER2 mRNA translation activity. To test this possibility, we performed the polysome profiling assay by sucrose gradient centrifugation and then measured the levels of HER2 mRNA by RT-PCR. As seen in Fig. 7A, both the monosomes and polysomes were detectable in the collected sample fractions of CT26/HER2 and CT26/HER2-A2 cells. For RT-PCR assay, RNAs were purified from the selected fractions (indicated by arrows). As shown in Fig. 7B, HER2 mRNAs were present in the monosome (ⓐ) and polysomes (ⓑ, ⓒ, ⓓ) of wild type CT26/HER2 cells, whereas they were not present in the monosome ⓔ and polysomes (ⓕ, ⓖ, ⓗ) of CT26/HER2-A2 cells. However, control GAPDH (glyceraldehyde 3-phosphate dehydrogenase) mRNAs were present in all of the tested monosomes and polysomes of CT26/HER2 and CT26/HER2-A2 cells. Thus, these results suggest that CT26/HER2-A2 cells may lose antigen expression by suppressing the translation activity of HER2 mRNAs at the post-transcriptional level, thus evading antitumor immune surveillance.

## Discussion

In the present study, we observed that CT26/HER2 tumor cells acquired antitumor CTL resistance through the loss of antigen expression in the prophylactic model. Although most immunized animals were protected from tumor formation, a few developed tumors particularly when they were challenged with a high number of tumor cells. These findings suggest that when a high number of tumor cells are injected into an animal with tumor antigen-specific adaptive immunity, they tend to easily acquire resistance to Ag-specific CTLs. This may be due to the possibility that a high number of tumor cells likely contain more heterogeneous tumor cell populations, some of which can be easily altered to a cell without tumor antigen expression, conferring resistance to tumor antigen-specific CTLs. We observed that tumor cells obtained from tumor-formed animals expressed MHC class I molecules, but not HER2 antigens, on the cell surface, and displayed antitumor immune resistance by forming tumors in HER2-immune mice. Moreover, a lack of HER2 protein expression was further confirmed by Western blot and IFN-γ release assays, as well as ELISA. These results, showing antigen loss as a way to evade antitumor immunity, are in agreement with our recent observation in a MC32 prophylactic tumor model^15^. Contrary to this, antigen loss was not associated with tumor immune evasion in the MC32 and CT26/HER2 therapeutic tumor models^9,12^. Taken together, our findings suggest that tumor cells utilize different strategies to escape tumor antigen-specific CTL immunity, which appear to rely on the status of CTL induction in animals when tumor cells are injected.

In the current study, we demonstrated that CT26/HER2-A2 tumor cells had a defect in antigen expression at the post-transcriptional level. This is based upon our results that the tumor cells expressed HER2 mRNAs but not HER2 proteins. DNA sequencing analysis revealed that a whole HER2 gene, as well as its upstream and downstream sequences had no genetic mutation in the immune-resistant cells, which were also unable to induce IFN-γ production when used as stimulating agents for immune cells from HER2-immune mice. Moreover, this genetic assay confirms that the two cell types, CT26/HER2-A1 and -A2 are indeed derived from wild type CT26/HER2 cells with retroviral integration of HER2 genes. Taken together, this result suggests that antigen loss might be resultant from the post-transcriptional regulation of antigen expression. When CT26/HER2-A2 cells were treated with proteasome and lysosome protease inhibitors, they were still unable to recover the ability to express HER2 protein and stimulate immune cells for IFN-γ production. In addition, there was no significant difference in the expression levels of HSP90 between CT26/HER2 cells and CT26/HER2-A1/-A2 cells. Taken together, these data indicate that the proteasome and lysosome protein degradation pathways are not associated with the loss of HER2 protein expression in these immune-resistant tumor cells. Here it is also notable that CT26/HER2-A1 and -A2 cells expressed MHC class I and PD-L1 molecules on the cell surface more than CT26/HER2 cells. In this case, however, it is unlikely that increased levels of MHC class I and PD-L1 expression may be associated with tumor immune evasion. This is because CT26/HER2-A1 and -A2 cells are not recognized by Ag-specific T cells due to the loss of HER2 antigen expression.

It has been reported that RNA-binding proteins (RBPs) and miRNAs act as post-transcriptional regulators. For instance, RBPs, which are composed of various proteins, small nuclear RNAs, and miRNAs, regulate the fates of mRNA by binding to mRNAs through their RNA binding domains (reviewed in^27,28^). In contrast, miRNAs, small non-encoding RNAs, bind to 3′ untranslated region of target mRNAs, leading to mRNA destabilization or repression of translation (reviewed in^29^). In addition, acquisition of antitumor drug resistance is regulated by RBPs and miRNAs in tumor cells, which alter the stability and translation of mRNAs coding for proteins involved in cell survival and regulation (reviewed in^26^). Using the Affymetrix GeneChip® miRNA 4.0 Array (Macrogen, Seoul, Korea), we found 184 miRNAs whose expression was up- or down-regulated by more than 1.5-fold in CT26/HER2-A2 cells as compared with CT26/HER2 cells (data no included). However, we were unable to recover HER2 expression by treating these immune-resistant cells with chemotherapeutic drugs (5-FU, padexol, gemcitabine, cisplatin, bleomycin, holoxan). In our subsequent polysome profiling assay, on the other hand, we observed that HER2 mRNA was not present in the monosomes and polysomes of CT26/HER2-A2 cells (lacking HER2 expression), while HER2 mRNA was present in the monosomes and polysomes of CT26/HER2 cells. A similar finding was observed in CT26/HER2-A1 cells (data not included). These results suggest that tumor cells can inhibit the translation activity of their antigen mRNAs, leading to loss of antigen expression. In this context, it is likely that the post-transcriptional regulation of antigen gene expression is one way by which tumor cells counter an immune selective pressure. It is also possible that for immune evasion, tumor cells can regulate the expression of endogenous antigens in a manner similar to the observed transgenic HER2 antigens as both of these antigen types act as a target of immune attack. Thus, these data collectively show that tumor cells may lose their antigen expression by suppressing the translation activity of their antigen mRNAs at the post-transcriptional level, thus escaping antitumor immune surveillance.

In conclusion, these studies showed that tumor cells escape antitumor immune surveillance by losing antigen expression in the protective tumor model of CT26/HER2 tumor cells. RT-PCR, qRT-PCR, PCR, Western blot and DNA sequencing assays demonstrated that the tumor cells lose HER2 antigens at the post-transcriptional level. During this process, tumor cells inhibited the translation activity of their antigen mRNAs, leading to the loss of antigen expression. Thus, these data show that tumor cells tend to lose antigen expression by suppressing the translation activity of their antigen mRNAs, thus evading antitumor immunity.

## Methods and Material

### Animals and cells

Six week-old female BALB/c mice were purchased from Daehan Biolink (Chungbuk, Korea). The mice were cared for under the guidelines of the Kangwon Institutional Animal Care and Use Committee-approved protocols (KW-130419-1). CT26/HER2 cells expressing HER2 proteins are colon cancer cell lines of a BALB/c mouse origin^30^. They were constructed by use of a retroviral construct containing the DNA coding for the human HER2 gene^30^. The cell line was kindly provided from H.J. Hong (Kangwon National University, Korea). The cells were maintained in cDMEM media (supplemented with 10% FBS [fetal bovine serum], 1% L-glutamine, 1% penicillin/streptomycin).

### Reagents and treatment of mice and cells

For intramuscular (IM)-electroporation (EP) delivery, mice were injected intramuscularly (i.m.) with 50 µg of HER2 DNA vaccines (pVAX1-HER2) per mouse in a final volume of 50 µl of phosphate-buffered saline (PBS) using a 31-gauge needle (BD, Franklin Lakes, NJ). The injections were followed by EP at 0.2 V for 4 sec using Cellectra® of VGX International Inc./Inovio in accordance with the manufacturer’s protocol. The HER2 DNA vaccines coding for an extracellular part of HER2 proteins were kindly provided from W.Z. Wei (Wayne State University, Detroit, MI). Plasmid DNA was produced in bacteria and purified by endotoxin-free Qiagen kits according to the manufacturer’s protocol (Qiagen, Valencia, CA). For protein degradation inhibition assay, MG132 (proteasome inhibitor) and chloroquine diphosphate salt (lysosomal degradation inhibitor) were purchased from Enzo Life Sciences (Farmingdale, NY) and Sigma-Aldrich (Saint Louis, MO), respectively.

### IFN-γ Assay

A 1 ml aliquot containing 6 × 10^6^ splenocytes was added to each well of 24-well plates containing 5 × 10^5^ tumor cells, which had been exposed to ultraviolet (UV) light for 3 h prior to immune cell stimulation. After 2 days of incubation at 37 °C in 5% CO_2_, cell supernatants were isolated and used to analyze IFN-γ levels, which was performed with commercial cytokine kits (BD Biosciences, San Jose, CA) and by adding the extracellular fluids to IFN-γ-specific enzyme-linked immunosorbent assay (ELISA) plates.

### Polysome profiling analysis

Tumor cells were grown to 80–90% confluency and harvested to obtain cell lysates in accordance to the polysome profiling analysis protocol^31^. Approximately 10 optical density (OD) amounts of the cell lysates were loaded on the sucrose gradient layer. Sucrose gradients (from 10% to 50%) were prepared in accordance to the polysome profiling analysis protocol^31^. The samples were centrifuged at 35,000 rpm for 2 h at 4 °C using SW55Ti rotor in a Beckman Coulter. The samples were then fractionated by carefully collecting 100 μl from the top layer using a micropipette. The OD values of each fraction were read at 260 nm using a Nanodrop spectrophotometer (ACTGene, Piscataway, NJ) and graphed using the Microsoft Excel software.

### PCR, RT-PCR, qRT-PCR and DNA sequencing assays

For PCR assay, tumor cells were lysed and the genomic DNA was purified using the genomic DNA purification kit, in accordance with the manufacturer’s protocol (Bioneer, Daejon, Korea). Four hundred ng of the genomic DNA was reacted with primers for 35 cycles (95 °C for 20 sec, 60 °C for 20 sec, 72 °C for 20 sec). The primers for HER2 (forward primer: 5′-CCTCTGACGTCCATCGTCTC-3′ and reverse primer: 5′-CGGATCTTCTGCTGCCGTCG-3′) were previously reported^32^. Mouse GAPDH primers were previously tested^15^. The final DNA product was separated by gel electrophoresis on a 1.5% agarose gel. For RT-PCR assay, total RNA was isolated from tumor cells or sucrose gradient factions using TRIzol reagents (Sigma-Aldrich). The RNA was treated with RNase-free DNase in accordance with the manufacturer’s protocol (Promega, Madison, WI) and then used for cDNA synthesis using AMPIGENE cDNA synthesis kit in accordance with the manufacturer’s protocol (Enzo Life Sciences). The cDNA was then used as a template for PCR amplification of HER2 and GAPDH using HiPi PCR premix kit in accordance with the manufacturer’s protocol (Elpisbiotech, Daejeon, Korea). For qRT-PCR assay, the cDNA and genomic DNAs were reacted using HiPi real-time PCR 2x Master Mix (SYBR Green, ROX) according to the manufacturer’s protocol (Elpisbiotech). The primers for HER2 and GAPDH were used as above under the PCR conditions (40 cycles; 95 °C for 20 sec, 60 °C for 20 sec, 72 °C for 20 sec). The HER2 mRNA levels were determined by using ABI PRISM 7000 Sequence Detection System (Applied Bio-system) with target-specific primers. The fold changes for HER2 mRNA were calculated using 2^−ΔΔCt^ method as previously described^33^. In the case of HER2 DNA, Ct values were compared. For DNA sequencing assay, tumor cells were lysed and genomic DNA was isolated as described above. Four hundred ng of the genomic DNA was reacted with primers for 35 cycles (95 °C for 30 sec, 60 °C for 30 sec, 72 °C for 45 sec). The primers for early HER2 gene regions (primer ⓐ; 5′-TTTTTGTGGCCCGACCTGAG-3′ and primer ⓑ 5′-CAAGAGGGCGAGGAGGAG-3′) were designed to have a 0.8 kb length of both early HER2 genes (1–50 nucleotides) containing translation start codon (ATG) plus their upstream sequences up to the MMLV ψ regions. The primers for HER2 genes (1–999 nucleotides) were 5′-ATGGAGCTGGCGGCCTTGTG-3′ (primer ⓒ) and 5′-CTTCTCACACCGCTGTGTTCC-3′ (primer ⓓ). The primers for HER2 genes (950–2030 nucleotides) were 5′-TGCACAACCAAGAGGTGACA-3′ (primer ⓔ) and 5′-CGCTTGATGAGGATCCCAAAG-3′ (primer ⓕ), The primers for HER2 genes (1972–2980 nucleotides) were 5′-GTGGTTGGCATTCTGCTGGT-3′ (primer ⓖ) and 5′-AGTCCTCATTCTGGATGACCA-3′ (primer ⓗ). The primers (primer ⓘ; 5′-GGAGTTGGTGTCTGAATTCTC-3′ and primer ⓙ; 5′-ACCCTAACTGACACACATTCC-3′) were designed to sequence late HER2 genes (2910–3768) and their downstream sequences up to the SV40 promoter region. HER2 cDNA nucleotides (NCBI Reference Sequence: NM_004448.3) and the nucleotide sequences upstream and downstream of HER2 genes (pBABE vector, Addgene, Cambridge, MA) were tested. The amplified DNA was run on a 1.5% agarose gel and then gel-purified in accordance with the manufacturer’s protocol (MP Biomedicals, Solon, OH). The purified DNA was sequenced using the above-described primers by Bionics, Seoul, Korea.

### Western blot assay

Tumor cells were lysed with RIPA lysis buffer containing protease inhibitor cocktail. Thirty μg of cell lysates were tested using sodium dodecyl sulfate (SDS)-polyacrylamide gel electrophoresis (PAGE) as previously described^34^. Anti-HER2 Abs were purchased from Sino Biological Inc. (Beijing, China). Anti-HSP90 and anti-mouse GAPDH Abs were purchased from Cell Signaling Technology, Inc.

### Fluorescence-activated cell sorting (FACS) analysis

Tumor cells were reacted at 4 °C for 30 min with phycoerythrin (PE)-labeled Abs specific for Fas-L, PD-L1, CD73 and CD80, as well as fluorescein isothiocyanate (FITC)-labeled Abs specific for MHC class I (H-2K^d^) in parallel with PE/FITC-labeled isotype control Abs for FACS analysis. These antibodies were purchased from BD Biosciences (San Diego, CA). For the detection of HER2 antigens, the cells were reacted with 2 μl sera of mice that had been immunized twice with HER2 DNA vaccines by IM-EP, followed by reaction with FITC-labeled anti-mouse IgG Abs (BD Biosciences). Finally, the cells were tested using a flow cytometer (BD Biosciences).

### Tumor cell challenge studies

Animals were challenged s.c. with 5 × 10^5^ to 1 × 10^6^ CT26/HER2 cells per mouse. For tumor forming ability assay, 3–5 × 10^5^ CT26/HER2, CT26/HER2-A1 and CT26/HER2-A2 cells per mouse were injected into each flank site of BALB/c mice. The tumor cells were grown in cDMEM, washed 2 times with PBS and injected into mice. The mice were monitored twice per week for tumor growth. The tumor growth was measured in mm using a caliper, and was recorded as mean diameter {longest surface length (a) and width (b), (a + b)/2}.

### Statistical analysis

Statistical analysis was performed by the independent *t* test and one-way ANOVA using the SPSS 17.0 software program. The values of the experimental groups were compared with the values of the control group. Any *p* values < 0.05 were considered to be significant.
